# 信迪利单抗致肺癌患者免疫性血小板减少1例并文献复习

**DOI:** 10.3779/j.issn.1009-3419.2023.102.32

**Published:** 2023-09-20

**Authors:** Jingjing CAI, Guangxia YANG, Xuemei ZHANG, Linlin LIU, Mei YAN

**Affiliations:** 272029 济宁，济宁医学院附属医院呼吸与危重症医学科; Department of Respiratory and Critical Care Medicine, Affiliated Hospital of Jining Medical University, Jining 272029, China

**Keywords:** 免疫检查点抑制剂, 信迪利单抗注射液, 免疫性血小板减少症, 肺肿瘤, 免疫相关不良事件, Immune checkpoint inhibitors, Sintilimab injection, Immune thrombocytopenia, Lung neoplasms, Immune-related adverse events

## Abstract

免疫检查点抑制剂（immune checkpoint inhibitors, ICIs）在肺癌的治疗中显示出独特优势，使肺癌的治疗进入了免疫治疗时代，但是ICIs也会出现不良反应，免疫治疗导致的血液系统毒性发病率并不是很高，其中免疫治疗导致的血小板减少是一种罕见的不良反应。我们报道1例使用ICIs导致血小板减少患者的病例资料，并对ICIs相关血小板减少进行文献复习，讨论相关临床特点、可能的机制及最佳治疗方式。

## Abstract (en)

Immune checkpoint inhibitors (ICIs) show unique advantages in the treatment of lung cancer, making the treatment of lung cancer enter the era of immunotherapy, but ICIs will also have adverse reactions, and the incidence of immune-induced hematological toxicity is not very high. Immunotherapy-induced thrombocytopenia is a rare adverse event.We report one case of thrombocytopenia induced by ICIs and review the literature on thrombocytopenia associated with ICIs and discuss the clinical features, possible mechanisms, and optimal treatment.

## 1 病例资料

患者，男，58岁，既往“2型糖尿病”；吸烟：40支/日，40年，有少量饮酒史；2021年9月10日因“咳嗽、痰中带血2月余”入院。入院查体无明显异常。入院后相关检查示：肿瘤标志物：癌胚抗原（carcinoembryonic antigen, CEA）8.46 ng/mL，细胞角蛋白19片段（cytokeratin 19 fragment, CYFRA21-1）9.89 ng/mL，神经元特异性烯醇化酶（neuron-specific enolase, NSE）25.91 ng/mL。胸部增强计算机断层扫描（computed tomography, CT）示：右肺中叶-右肺门占位性病变，考虑肺癌并阻塞性肺炎、肺不张伴右肺门及纵隔内淋巴结肿大（图1），排除禁忌后行CT引导下肺穿刺活检，病理回示：（右肺中叶穿刺组织）非小细胞癌（non-small cell lung cancer, NSCLC），免疫组化倾向于腺癌。免疫组化：细胞角蛋白7（cytokeratin 7, CK7）（+），甲状腺转录因子1（thyroid transcription factor-1, TTF-1）（+），天冬氨酸蛋白酶A（novel aspartic proteinase A, Napsin A）（+），P63（-），P40（-），CK5/6（-），Ki-67（+，40%）。颅脑平扫+增强磁共振（2021-09-15）未见明显异常，全身骨显像（2021-09-16）未见骨转移，颈部淋巴结彩超（2021-09-14）示右侧锁骨上区多发异常肿大淋巴结，结合病史，考虑转移灶。腹部彩超+肾上腺彩超（2021-09-14）示肝右叶囊肿、右肾囊肿、双侧肾上腺区未见明显异常。诊断为右肺腺癌（cT2aN3M0，IIIB期），无手术指征，基因检测：基于本次检测，查见Kirsten大鼠肉瘤病毒癌基因同源物（Kirsten rats arcomaviral oncogene homolog, KRAS）基因2号外显子G12C突变。程序性死亡配体1（programmed cell death ligand 1, PD-L1）检测：肿瘤细胞阳性比例分数（tumor proportion score, TPS）为80%，美国东部肿瘤协作组（Eastern Cooperative Oncology Group, ECOG）体能状态评分为1分，患者家属拒绝化放疗，排除禁忌后于2021年9月30日给予单药信迪利单抗注射液200 mg，静脉滴注，q3w，共治疗9个周期，经过治疗后患者未再出现痰中带血，咳嗽较前减轻，根据实体瘤疗效评价标准（Response Evaluation Criteria in Solid Tumor, RECIST）1.1版评价为部分缓解（partial response, PR），无进展生存期（progression-free survival, PFS）目前已达9个月（图1），在9个周期信迪利单抗治疗后院外未复查血常规，在30 d复查血常规示：血小板25×10^9^/L，白细胞和血红蛋白水平正常，在此期间患者没有出现瘀伤、瘀点，追问病史既往无自身免疫或凝血功能障碍史，完善抗核抗体定量、抗核抗体谱测定未见异常，抗血小板抗体弱阳性。完善骨髓穿刺+活检术：骨髓红巨系增生活跃伴巨核产板不良：粒细胞比例减低，部分胞浆颗粒增多（图2），根据患者病史、骨髓穿刺报告、血液相关检查，临床诊断为信迪利单抗诱导的免疫性血小板减少症（immune thrombocytopenia, ITP），给予注射用人白介素11（interleukin-11, IL-11）1.5 mg皮下注射，qd，8 d，地塞米松磷酸钠注射液10 mg静推，qd，4 d，出院后序贯口服醋酸泼尼松片30 mg，qd，1周后减量至20 mg，然后每周减5 mg直至停药，总共疗程6周，期间多次复查血小板逐渐恢复正常（图3）。根据中国临床肿瘤学会（Chinese Society of Clinical Oncology, CSCO）指南免疫检查点抑制剂（immune checkpoint inhibitors, ICIs）相关的毒性管理及《常见不良反应术语评定标准4.03（Common Terminology Criteria for Adverse Events v4.03, CTCAE-4.03）》，患者血小板减少分级为3级，暂停免疫治疗，密切随访及治疗，如果恢复到1级可继续免疫治疗，考虑重启免疫治疗风险高，建议患者放化疗，患者仍拒绝，于2022年8月5日重启免疫治疗，重启治疗2个周期后再次出现血小板减少，遂终止免疫治疗。

**图1:**
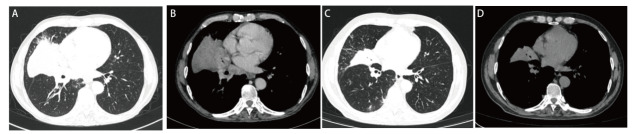
患者治疗前后胸部CT改变。A、B：信迪利单抗治疗前右肺中叶-右肺门见组织团块影；C、D：信迪利单抗治疗9个周期后，右肺占位较前明显缩小。

**图2:**
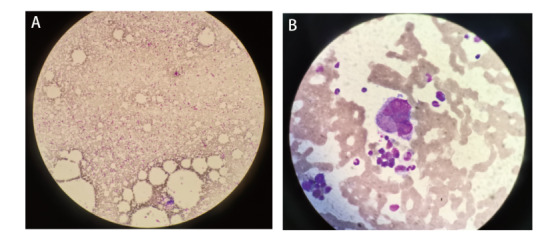
患者骨髓涂片细胞学结果。A：有核细胞增生活跃，2 cm×3 cm面积见巨核细胞208个，粒系38.5%，红系52.0%，分类25个巨核细胞，其中幼稚巨核细胞1个，颗粒巨核细胞17个，成熟产板巨核细胞1个，裸核巨核细胞3个，巨核细胞形态未见明显异常（低倍镜，×100）；B：仅数个血小板释放巨核细胞（油镜，×1000）。

**图3:**
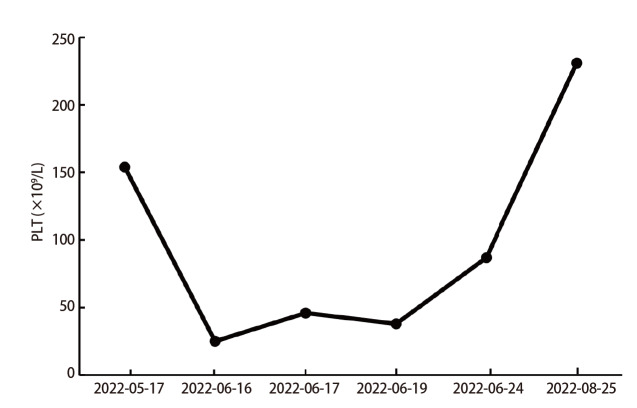
住院治疗期间血小板计数的变化

## 2 讨论

以“免疫性血小板减少、免疫检查点抑制剂”和“immune thrombocytopenia, immune checkpoint inhibitor”为关键词分别在万方数据库、中国知网和PubMed数据库进行检索并对相关文献进行复习。共纳入8例免疫治疗导致的血小板减少患者，年龄34-82岁，男女比例为3:1。其中7例患者使用纳武利尤单抗，1例使用卡瑞利珠单抗，在免疫治疗1-8个周期出现ITP，血小板最低为2000/μL，治疗上予以激素、免疫球蛋白及血小板生成素受体激动剂，其中5例好转，3例死亡（见表1）。对于NSCLC患者，ICIs通常比细胞毒性药物更安全，耐受性更好。通过相关文献复习，在使用1-8个周期ICIs后会出现ITP，本例患者在使用信迪利单抗9个周期后出现ITP。

**表1:** 非小细胞肺癌患者免疫检查点抑制剂诱发免疫相关性血小板减少症的报告病例

Author	Age (yr)	Gender	Immune checkpoint inhibitors	Treatment cycle	Minimum platelet value	Treatment method	Prognosis
Mori, et al.^ [1]^	77	Male	Nivolumab	1	2000/μL	Hormone	Return to normal
Bagley, et al.^ [2]^	34	Male	Nivolumab	8	33,000/μL	Thrombopoietin receptor agonists	Turn for the better
Karakas, et al.^ [3]^	78	Male	Nivolumab	6	5000/μL	Hormone	Death
Jotatsu, et al.^ [4]^	62	Male	Nivolumab	2	1600/μL	Hormone	Turn for the better
Tokumo, et al.^ [5]^	56	Female	Nivolumab	3	19,000/μL	Hormone+immunoglobulin	Death
Hasegawa, et al.^ [6]^	82	Female	Nivolumab	2	2000/μL	Hormone+immunoglobulin+thrombopoietin receptor agonists	Death
Khorasanchi, et al.^ [7]^	67	Male	Nivolumab	1	1000/μL	Hormone+immunoglobulin+Rituximab	Turn for the better
Xu, et al.^[8]^	60	Male	Carrilizumab	1	2×10^9^/L	Hormone+immunoglobulin	Turn for the better

ICIs目前已被批准用于治疗各种恶性肿瘤，包括肺癌。ICIs能够重新激活免疫系统，启动肿瘤杀伤，而T细胞过度激活会导致各种免疫相关不良反应的发生。ICIs相关的毒性包括皮肤相关不良反应、结肠炎、肝炎、内分泌系统不良反应、神经系统不良反应和罕见的血液系统不良反应^[9]^。一项对2360例接受ICIs治疗的黑色素瘤患者的回顾性研究^[10]^显示，低于1%的患者出现血小板减少，其中大多数患者血小板自行恢复正常。有关SHR-1210（Camrelizumab）的I期临床研究^[11]^显示，其中3-4级毒性发生率为2%；血小板减少症为1%，无3-4级免疫不良反应发生。ITP一旦发生，如果达到2级及以上需要暂时终止抗肿瘤治疗，有些甚至可能危及生命，因此临床医师必须认识到这种相对罕见的ITP。

ITP是一种以T细胞失调为特征的自身免疫性疾病，其特征是产生针对血小板抗原的自身抗体。程序性死亡受体1（programmed cell death 1, PD-1）和PD-L1是共信号分子，PD-1途径的主要作用是抑制自身反应性T细胞并预防自身免疫性疾病。Birtas等^[12]^分别对21名健康人和67例ITP患者进行血清可溶性PD-1和PD-L1检测，发现PD-1和PD-L1在ITP患者中均降低，并且与血小板计数存在正相关，在没有PD-1抑制监管下，持续激活的T细胞可能会导致ITP的炎性反应。血清可溶性PD-1可能导致PD-1/PD-L1信号通路的功能障碍，其表达水平与ITP患者的严重程度有关。使用血清可溶性PD-1去激活PD-1/PD-L1可以恢复ITP患者T辅助细胞（helper T cell, Th）1/Th2和辅助性T细胞（regulatory T cells, Treg）/Th17细胞亚群的失衡，但抗PD-1可能通过增强干扰素-γ（interferon-γ, IFN-γ）的产生而加重疾病^[13]^。

ITP是一种排除性诊断，由于缺乏特异性检查以及其鉴别诊断范围广，且ICIs诱导的ITP的发生是罕见的，在诊断免疫导致的血液系统不良反应时应注意排除这些相关因素。本例患者单药使用信迪利单抗，没有使用全身化疗及放疗，追问病史既往无自身免疫或凝血功能障碍史，血糖控制良好，完善抗核抗体定量、抗核抗体谱测定未见异常，抗血小板抗体弱阳性，完善骨髓穿刺检查：骨髓红巨系增生活跃伴巨核细胞产板不良：粒细胞比例减低，部分胞浆颗粒增多，复查胸部CT肿瘤稳定，使用糖皮质激素治疗有效，在重启免疫治疗后再次出现血小板减少，综合以上，患者最终诊断为信迪利单抗诱导的ITP。因此肺癌患者在进行ICIs治疗时要注意监测血常规，还需要相关临床研究来进一步确定患者特异性的危险因素。

Competing interests

The authors declare that they have no competing interests.
